# Identification, Expression, and Regulation of an Omega Class Glutathione S-transferase in *Rhopalosiphum padi* (L.) (Hemiptera: Aphididae) Under Insecticide Stress

**DOI:** 10.3389/fphys.2018.00427

**Published:** 2018-04-20

**Authors:** Balachandar Balakrishnan, Sha Su, Kang Wang, Ruizheng Tian, Maohua Chen

**Affiliations:** State Key Laboratory of Crop Stress Biology for Arid Areas and Key Laboratory of Crop Pest Integrated Pest Management on the Loess Plateau of Ministry of Agriculture, Northwest A&F University, Yangling, China

**Keywords:** glutathione S-transferase, *Rhopalosiphum padi*, insecticide detoxification, omega class, gene expression

## Abstract

Glutathione S-transferases (GSTs) play an essential role in the detoxification of xenobiotic toxins in insects, including insecticides. However, few data are available for the bird cherry-oat aphid, *Rhopalosiphum padi* (L.). In this study, we cloned and sequenced the full-length cDNA of an omega GST gene (*RpGSTO1*) from *R. padi*, which contains 720 bp in length and encodes 239 amino acids. A phylogenetic analysis revealed that RpGSTO1 belongs to the omega class of insect GSTs. RpGSTO1 gene was highly expressed in transformed *Escherichia coli* and the protein was purified by affinity chromatography. The recombinant RpGSTO1 displayed reduced glutathione (GSH)-dependent conjugating activity toward the substrate 1-chloro-2, 4-dinitrobenzene (CDNB) substrate. The recombinant RpGSTO1 protein exhibited optimal activity at pH 7.0 and 30°C. In addition, a disk diffusion assay showed that *E. coli* overexpressing RpGSTO1 increased resistance to cumene hydroperoxide-induced oxidative stress. Real-time quantitative PCR analysis showed that the relative expression level of *RpGSTO1* was different in response to different insecticides, suggesting that the enzyme could contribute to insecticide metabolism in *R. padi*. These findings indicate that RpGSTO1 may play a crucial role in counteracting oxidative stress and detoxifying the insecticides. The results of our study contribute to a better understanding the mechanisms of insecticide detoxification and resistance in *R. padi*.

## Introduction

Glutathione S-transferases (GSTs; EC 2.5.1.18) are a family of multifunctional phase II enzymes that play a crucial role in the detoxification of many exogenous and endogenous xenobiotics compounds and have been widely found in almost all living organisms (prokaryotic and eukaryotic) (Booth et al., 1961; Tu and Akgül, 2005; Li et al., 2007). The enhanced metabolic capability of detoxification enzymes, such as carboxylesterase (CarE), cytochrome P450 monooxygenases (P450) and GSTs are important for insecticide resistance (Rufingier et al., 1999; Puinean et al., 2010; Cui et al., 2015). The major function of GSTs is conjugation of electrophilic compounds with the thiol group of reduced glutathione (GSH), thus making them less toxic, more soluble and easier to excrete from the cell (Enayati et al., 2005; Ketterman et al., 2011). Cytosolic insect GSTs can be classified into six major classes: delta, epsilon, omega, sigma, theta, and zeta; there are also several unclassified genes (Ranson et al., 2001). Different classes of GSTs can be distinguished based on their primary amino acid sequences; identity is approximately 50% within a class and less than 30% among different classes (Sheehan et al., 2001; Mannervik et al., 2005). The omega class of GSTs (GSTO) is one of the largest GST subfamilies, with multiple functions identified in various species. GSTOs have unique structures and play essential physiological roles that differ from other GST classes (Meng et al., 2014). GSTOs are ubiquitous across taxa and play an essential physiological role including detoxifying insecticides (Chen and Zhang, 2015; Wu and Hoy, 2016). The recent studies indicate that GSTOs are also involved in oxidative response (Meng et al., 2014). However, the mechanisms involved the GSTOs effect still need further clarification. The first GSTO was identified through a bioinformatics analysis of expressed sequence tags in humans (Board et al., 2000). GSTOs have since been found in plants, yeast, bacteria and insects (Dixon et al., 2002; Garcerá et al., 2006; Walters et al., 2009; Xun et al., 2010). In GSTOs, a novel cysteine residue (Cys) is present in the active site, whereas GSTs from other classes have canonical serine and tyrosine residues (Caccuri et al., 2002). Insect GSTs display different substrate specificities, catalytic activities and have unique N-terminal and C-terminal extensions that are not observed in the other GST classes (Board, 2011). As GSTs can play roles in detoxification of various insecticides, a change in the GST activity is one mechanism of metabolic resistance to insecticides (Ranson and Hemingway, 2005; Li et al., 2007).

Aphids are common phloem-feeding pests found worldwide, and they damage plants by removing nutrients (Rabbinge et al., 1981). The bird cherry-oat aphid, *Rhopalosiphum padi* (L.) (Hemiptera: Aphididae), is a serious wheat pest in China (Wang et al., 2006). It can significantly reduce grain yields (*Triticum aestivum* L.) (Kieckhefer and Gellner, 1992; Blackman and Eastop, 2000) and is also an important vector for the barley yellow dwarf virus, which infects and damages wheat crops (Watson and Mulligan, 1960). Insecticides are stress factors that can affect many physical and biochemical process in insects. Insect populations have increased over time due to acquisition of insecticide resistance (Bass et al., 2014).

Here, we report the identification and classification of an omega class GST gene (*RpGSTO1*) from *R. padi*. The recombinant protein, RpGSTO1, was expressed in *Escherichia coli* cells. The biochemical properties of the purified recombinant GST protein were characterized. The transcriptional patterns of *RpGSTO1* following exposure to various concentrations of β-cypermethrin, isoprocarb, malathion, and sulfoxaflor were analyzed. The potential roles of the RpGSTO1 fusion protein in antioxidant defense were also investigated. Our results provide valuable insight into the function of RpGSTO1 in the stress response to insecticides.

## Materials and Methods

### Insects

*Rhopalosiphum padi* was collected from a wheat field in Gansu Province of China in 2013, and used to establish a colony on seedlings of wheat (cultivar “Xiaoyan 22”) in mesh cages (41 cm × 41 cm × 41 cm) in the laboratory. The colony was reared under regulated conditions (23 ± 1°C temperature, 70% relative humidity and 16 h light/8 h dark photoperiod) without microbial infection and without insecticide exposure (Wang et al., 2016).

### RNA Extraction and cDNA Synthesis

Total RNA was extracted from 15 apterous adult aphids using TRIzol reagent (Invitrogen, Carlsbad, CA, United States) according to the manufacturer’s instructions and treated with RNase-Free DNaseI (Takara, Kyoto, Japan) to remove genomic DNA contamination. The purity of the extracted RNA was determined by agarose gel electrophoresis, and the concentration was checked using a biophotometer (Eppendorf Biophotometer Plus, Eppendorf, Germany). First-strand complementary DNA (cDNA) was synthesized from 2 μg total RNA using M-MLV reverse transcriptase cDNA Synthesis Kit (Promega, Madison, WI, United States) according to the manufacturer’s instructions. The cDNA was stored at -80°C prior to use as the template for PCR in subsequent gene cloning procedures.

### Identification and Gene Cloning of Omega Glutathione S-Transferase Gene From *R. padi*

Using the published *R. padi* transcriptome data (Duan et al., 2017), sense and antisense primers were designed using Lasergene Primerselect (DNASTAR Inc, Madison, WI, United States) to amplify the full-length coding region for the omega GST gene, *RpGSTO1*. The amplification reaction mix contained 4 mM MgCl_2_, 100 μM dNTPs, 0.4 μM of forward and reverse primers, 2 units of Taq DNA polymerase (5 U/μL, Sangon Biotech Co., Ltd., Shanghai, China) and 1 μL of template DNA. Amplification occurred under the PCR conditions of 95°C for 3 min followed by 35 cycles of 95°C for 30 s, 55°C for 30 s, 72°C for 45 s and a final 5 min at 72°C. The PCR product was verified on 1% (*w*/*ν*) agarose gel and visualized after staining with SYBR green using an imaging instrument (Sagecreation Science Co., Beijing, China). The target GST gene product was purified using gel extraction kit (Promega, Madison, WI, United States). The purified PCR product was then ligated to the pGEM-T Easy Vector (Promega, Madison, WI, United States) and transformed into *Escherichia coli* DH5α competent cells (Takara, Kyoto, Japan). The transformants were selected on LB agar plates containing 50 μg/mL kanamycin grown overnight at 37°C. Five independent colonies were sequenced in both directions using an Applied Biosystems 3730 automated sequencer (Applied Biosystems, Foster City, CA, United States) at Sangon Biotech Co., Ltd. (Shanghai, China).

### Sequence Identity and Phylogenetic Analysis

The deduced amino acid sequence for *RpGSTO1* was determined using the NCBI open reading frame (ORF) finder website^1^. The ExPASy tool^2^ was used to predict the theoretical isoelectric point (pI) and molecular weight of the predicted protein. Sequence similarity was determined by aligning sequences with ClustalX (Chenna et al., 2003), and the file was converted for analysis using Molecular Evolutionary Genetic Analysis (MEGA) version 7.0 (Kumar et al., 2016). The phylogenetic tree was constructed using the neighbor-joining (NJ) method with pairwise deletion options, and the branch of the tree was evaluated using 1000 bootstrap replicates.

### Plasmid Construction and Recombinant Protein Expression

The *RpGSTO1* was amplified using a pair of primers containing restriction enzymes *Bam*HI and *Hind*III. The *Bam*HI restriction site was incorporated to sense primer, and *Hind*III restriction site was incorporated to antisense primer for double restriction digestion reaction. PCR fragments were purified using a gel extraction kit (Promega, Madison, WI, United States), cloned into the pGEM-T Easy vector and then digested with *Bam*HI and *Hind*III. The digested fragments were purified and ligated into the prokaryotic expression vector, pET-28a (Novagen, Merck, Germany), using a quick ligation kit (TaKaRa, Kyoto, Japan). The expression plasmid was transformed into *E. coli* BL-21 (DE-3) competent cells (Takara, Kyoto, Japan). The transformed cells were cultured in Luria-Bertani media containing 50 μL/mL kanamycin at 37°C with 220 rpm shaking until the OD_600_ reached 0.7. Then, isopropyl 1-thio-β-D-galactopyranoside (IPTG) was added to a final concentration of 1 mM and the culture was shifted to 30°C to induce the production of RpGSTO1. After incubation for 3 h, the cells were harvested by centrifugation at 10,000 rpm for 3 min. The cell pellet was washed with sterile water and then resuspended in 20 mM Tris-HCL buffer (pH 8.0) containing 0.5 M NaCl, 1 mg/mL of lysozyme, and 1 mM phenylmethanesulfonyl fluoride (PMSF). The expressed recombinant protein was analyzed by 12% (w/v) sodium dodecyl sulfate-polyacrylamide gel electrophoresis (SDS-PAGE), using a standard protein marker (PageRuler^TM^ Prestained protein ladder). Protein bands were visualized by Coomassie Brilliant Blue R250 staining.

### Recombinant Protein Purification and Western Blot Analysis

The recombinant RpGSTO1 cells were grown at 37°C in 100 mL Luria-Bertani media containing 50 μg/mL kanamycin until the optical density (OD) reached 0.8. Then, 0.5 mM IPTG was added and cells were grown at 25°C overnight with shaking at 180 rpm. The cells were harvested by centrifugation at 12,000 rpm for 3 min. The cell pellet was resuspended in lysis buffer (20 mM Tris-HCL, pH 7.4, 500 mM NaCl, 15% glycerol, and 1 mM PMSF). The cell lysate was subjected to centrifugation at 12,000 rpm for 10 min at 4°C to remove the cellular debris, and the supernatant was passed through a 0.45-nM syringe filter. The filtered protein extract was loaded onto a cOmplete His-Tag purification resin affinity chromatographic column (Roche Diagnostics GmbH, Mannheim, Germany). Non-target protein in the supernatant was removed with wash buffer (50 mM NaH_2_PO_4_, 300 mM NaCl and 20 mM imidazole, pH 8.0). The protein was eluted with a linear imidazole gradient of 50–250 mM in buffer. The eluted samples were desalted using a dialysis membrane in 50 mM sodium phosphate buffer, pH 7.4 for 24 h at 4°C. The protein purity was checked by 12% (w/v) SDS-PAGE and stained with Coomassie Brilliant Blue R250. The concentration of protein was measured using a BCA protein assay kit (Cwbiotech, Beijing, China), with bovine serum albumin as the standard.

After electrophoresis, proteins were transferred to a polyvinylidene fluoride membrane (PVDF) by immune blotting. After blotting, the membrane was blocked by incubation for 2 h at room temperature in a 5% bovine serum albumin (BSA) solution. Then, membrane was incubated overnight with primary 6-His monoclonal antibody (1:2000, v/v) at 4°C, and then membrane was washed in TBST [10 mM Tris-HCL, pH 8.0, 100 mM NaCl and 0.1% (w/v) Tween 20]. The membrane was then incubated with 1:5000 (v/v) horseradish peroxidase-conjugated anti-mouse IgG. After repeated washing with TBST, the membrane immersed with ECL detection reagents (BioRad, Hercules, CA, United States).

### Measurements of Enzyme Activity

RpGSTO1 activity was determined spectrophotometrically using 1–chloro-2, 4-dinitrobenzene (CDNB) and reduced glutathione (GSH) as standard substrates (Habig et al., 1974). Enzymatic activity is expressed as mol CDNB conjugated with GSH per min per mg of protein. The stock solution of CDNB was prepared in ethanol, and GSH was dissolved in 0.1 M sodium phosphate buffer. Each 300-μL reaction mixture contained 100 ng of RpGSTO1, 0.5 mM CDNB, 5 mM GSH in 0.1 M phosphate buffer. The optimum pH for RpGSTO1 activity was determined at 30°C, with pH at 5.0, 5.5, 6.0, 6.5, 7.0, 7.5, or 8.0. The thermostability of RpGSTO1 was determined by preincubation of the enzyme solution at 10, 20, 30, 40, 50, 60, or 70°C for 30 min prior to performing a residual activity assay at pH 7.0. These optimal pH and temperature experiments were conducted with fixed concentrations of CDNB (0.5 mM) and GSH (5 mM). The reaction was monitored by measuring absorbance at 340 nm with 15 s intervals using a TECAN^TM^ Infinite^®^ 200 PRO multimode micro-plate reader (𝜀340 = 9600 M^-1^cm^-1^). The reduced GSH concentration was held at 5 mM, while CDNB concentration was varied from 0.02 to 0.14 mM. The kinetic parameters (*K_m_* and *V_max_*) were determined by linear regression of double reciprocal plot. All assays were performed in quadruplicate and repeated three times with non-enzymatic controls for reference blanks.

### Disk Diffusion Assay

A disk diffusion assay was performed in according to Yan et al. (2013). The *E. coli* culture containing overexpressed RpGSTO1 was plated on Luria-Bertani agar plates and incubated at 37°C for 1 h. Cells with the pET-28a (+) were used as the controls and treated with the same conditions. Filter disks (6-mm diameter) were soaked with different concentrations of cumene hydroperoxide (0, 30, 50, 100, and 200 mM). All the disks were placed on the surface of the agar plates and incubated at 37°C for 24 h. The inhibition zones around the disks were measured. The assay was repeated three times, and statistical significance of the inhibition zone was calculated using the program JMP13 (SAS Institute-9.3, Cary, NC, United States).

### Real-Time qPCR Analysis of *RpGSTO1* Expression Under Different Insecticide Stress

The β-cypermethrin, isoprocarb, malathion, and sulfoxaflor (Yancheng Nongbo Bio-technology co., Ltd., Jiangsu, China) used in this study were of technical grade. Based on our previous bioassay results (Wang et al., 2017), two concentrations (LC_25_ and LC_50_) of each insecticide were used. The LC_25_ and LC_50_ concentrations were 0.7671 mg/L and 1.3082 mg/L for β-cypermethrin, 0.0372 mg/L and 0.0618 mg/L for isoprocarb, 1.4230 mg/L and 2.7048 mg/L for malathion, and 0.0674 mg/L and 0.1217 mg/L for sulfoxaflor, respectively. A previously reported leaf-dipping method was adopted for insecticide stress treatment (Wang et al., 2016). Wheat leaves with 50–60 apterous adult aphids were dipped in the two concentrations (LC_25_ and LC_50_) of each chemical for 10–15 s and then dried with the help of filter papers. Wheat leaves treated with solution in the absence of insecticide were used as the control. Three replicates were maintained at a constant temperature of 23 ± 1°C and photoperiod of 16:8 (L:D) h both during and after treatment, and the live aphids were collected at 12, 24, or 36 h post-treatment.

Total RNA was isolated from the live aphids (5 mg) collected at each treatment, and expression of *RpGSTO1* was analyzed. Total RNA extraction and cDNA synthesis were performed as described above. The real-time quantitative PCR (qPCR) reactions were conducted in a Rotor Gene Q Real Time Thermal Cycler (Qiagen, Hilden, Germany) using SYBR Green to detect the amplification signals. Primers for qPCR are listed in **Table 1**. The *β-Actin* and *EF-1α* (elongation factor 1α) genes were used as internal references to normalize target gene expression (Wang et al., 2016; Li et al., 2017). The reaction mixture consisted of 1 μL cDNA template, 0.8 μL of 10 μM forward/reverse primers, 10 μL 2X FastStart Essential DNA Green Master^TM^ (Roche, Shanghai, China) and 7.4 μL RNase-free water. Thermal conditions were as follows: initial denaturation at 95°C for 10 min, followed by 40 cycles of denaturation at 95°C for 15 s, annealing 58°C for 30 s and elongation for 72°C for 30 s. The real-time data were acquired by raising the temperature from 65°C to 95°C for 10 s at 0.5°C increments. Reactions for all samples were performed independently repeated triplicates. The relative expression levels were calculated using the comparative CT method (2^- Δ Δ C_T_^) (Livak and Schmittgen, 2001).

**Table 1:** Oligonucleotide primer pairs used in this study.

Primer name	Primer sequence (5′-3′)	Application
RpGSTO1-F	AATTATTCTCCGGGTCGTCAA	ORF amplification
RpGSTO1-R	AAGTGCAATGTTTTAGCCTCAAG	
rRpGSTO1-F	CGGATCCATGGCCACCAAACACT TGTCCAAA	Protein expression
rRpGSTO1-R	CGAAGCTTTTAAATGTCATAAGCA GGTAATCCA	
RpGSTO1-qF	CCAAAGGTGCTAGGCTCATT	qRT-PCR
RpGSTO1-qR	CTGTTCGTCGAGGAAGTCTG	
β-Actin -F	GCCCAATCCAAAAGAGGTAT	qRT-PCR Reference gene
β-Actin -R	TCAAAGGTGCTTCCGTTAGT	
qEF-1αF	GCTCTATTGGCTTTCACCTT	
qEF-1αR	GATGTAACTGCTGACTTCTTTC	

### Statistical Analysis

All statistical analyses were performed using SAS JMP13 (SAS Institute-9.3, Cary, NC, United States). The results are presented as the mean ± standard error from triplicate experiments, and data were analyzed using Student’s *t*-test for comparison of two means or one-way analysis of variance followed by Tukey’s test. The level of significance was set at *p* < 0.05 for all treatments. All the graphs were created using Prism 6.0 for windows (GraphPad, La Jolla, CA, United States)^3^.

## Results

### Identification and Characterization of *RpGSTO1* Gene

**FIGURE 1:**
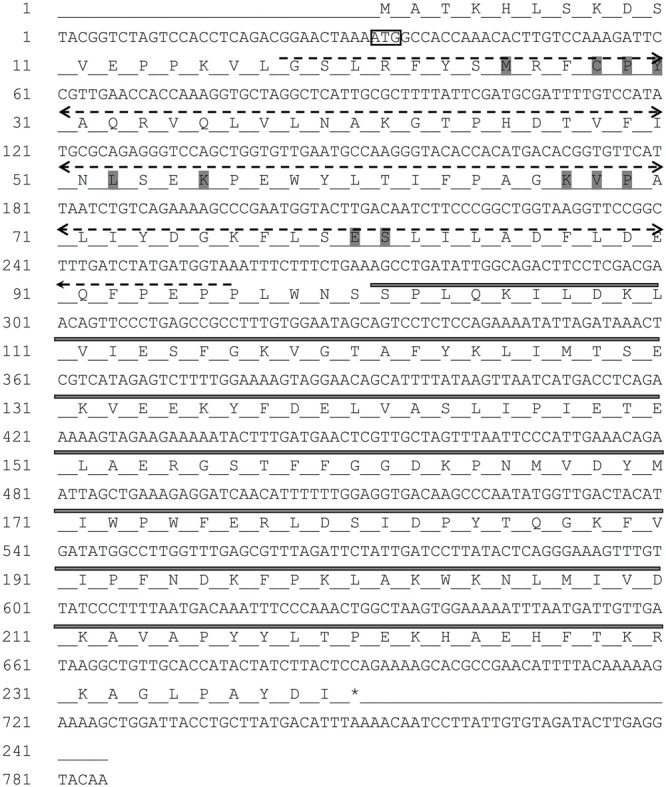
The nucleotide and deduced amino acid sequence of *RpGSTO1* gene. The start codon (ATG) is indicated with a box, and the termination codon (TAA) indicated with an asterisk. The putative glutathione binding region (G-site) is indicated in dash line, and the substrate binding region (H-site) in bold line. The conserved binding residues are highlighted in gray.

The full-length cDNA sequence of *RpGSTO1* gene was obtained from *R. padi* and deposited in GenBank (Accession Number: MG709032). The cDNA sequence of *RpGSTO1* is 785 bp long, which contains a 31-bp 5′ untranslated region (UTR), and a 34-bp 3′ UTR. The full length open reading frame (ORF) is 720 bp in length, encoding a 239-amino acid protein with a predicted molecular mass of 27.469 kDa and a theoretical pI of 6.13 (**Figure 1**).

### Phylogenetic Analysis of RpGSTO1 and Other Insect GSTs

**FIGURE 2:**
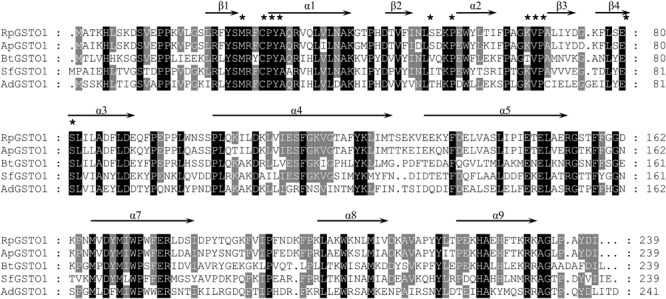
Amino acid sequence alignment of RpGSTO1 with the omega GSTOs from other insects. ApGSTO1, GSTO1 from *Acyrthosiphon pisum*, GenBank accession no: NP_001155757; BtGSTO1, GSTO1 from *Bemisia tabaci*, AST11637; SfGSTO1, GSTO1 from *Sogatella furcifera*, AFJ75814; and AdGSTO1, GSTO1 from *Apis dorsata*, XP_006623084. Identical amino acids are shaded in black and similar amino acids are shaded in gray. GSH binding residues are marked with asterisks.

**FIGURE 3:**
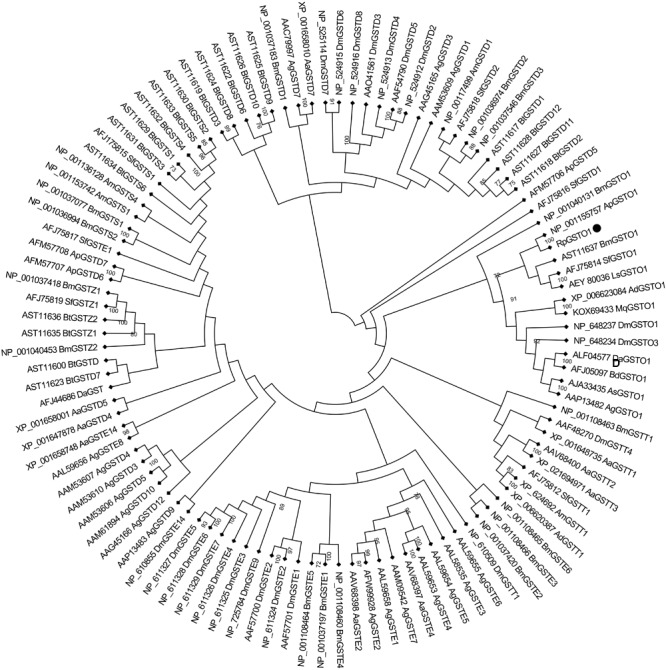
Phylogenetic relationships of omega RpGSTO1 with other insect GSTs. DM, *Drosophila melanogaster*; Bm, *Bombyx mori*; Sf, *Sogatella furcifera*; Ap, *Acyrthosiphon pisum*; Bt, *Bemisia tabaci*; Ad, *Apis dorsata*; Mq, *Melipona quadrifasciata*; Da, *Delia antique*; Ls, *Laodelphax striatella*; Bd, *Bactrocera dorsalis*; As, *Anopheles sinensis*; Ag, *Anopheles gambiae*; Aa, *Aedes aegypti*; and Am, *Apis mellifera*; The GenBank accession number is before each GST, and the capital letter D, E, O, S, T, and Z after each GST indicate the delta, epsilon, omega, sigma, theta, and zeta class of GST, respectively. The tree was constructed using neighbor-joining and bootstrap support values based on 1000 replicates by MEGA 7.0. RpGSTO1 is marked with a solid black circle.

The amino acid sequence of *RpGSTO1* has high identity with omega class GSTs from other insect species such as *Acyrthosiphon pisum* GSTO1 (GenBank: NP_001155757, 85% identity), *Bemisia tabaci* GSTO1 (GenBank: AST11637, 54% identity), *Sogatella furcifera* GSTO1 (GenBank: AFJ75814, 51% identity) and *Apis dorsata* (GenBank: XP_006623084, 45% identity) (**Figure 2**). A domain analysis revealed that the RpGSTO1 monomer includes 9 α-helics and 4 β-strands. The conserved residues of the insect cytosolic GSTs N-terminal and C-terminal domains were similar, and G-site implied common GSH-binding characteristics. RpGSTO1 shared the highest similarity with the pea aphid *A. pisum* GSTO1. A neighbor-joining phylogenetic tree was constructed using the MEGA tool with sequences of other insect cytosolic GSTs. The phylogenetic relationship analysis revealed that RpGSTO1 clustered together with the omega class GSTs. The GSTs from other classes (delta, epsilon, theta, omega, zeta, and sigma class) were generally clustered together in the tree (**Figure 3**).

### Expression and Purification of RpGSTO1

**FIGURE 4:**
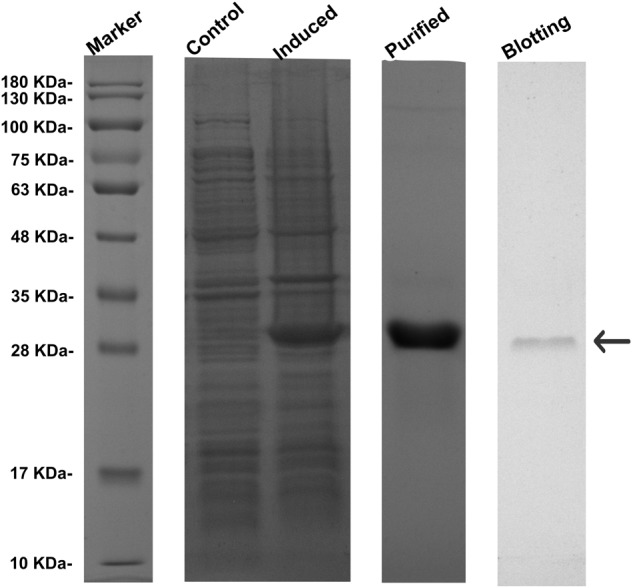
Expression profile and western blot analyses of RpGSTO1. Control, the crude extracts from the bacterial pellets without isopropyl β-D-1-thiogalactopyranoside (IPTG) induction. Purified, the recombinant RpGSTO1 expressed in *E. coli* BL-21 (DE-3) and purified using cOmplete His-Tag purification system. Blotting, the recombinant RpGSTO1 was identified using His-tag antibody. Protein molecular weight standards are used as a size marker.

Recombinant RpGSTO1 protein was successfully overexpressed in *E. coli*, as confirmed by SDS PAGE (**Figure 4**). The recombinant RpGSTO1 was in a soluble form and purified to homogeneity by His-Tag resin affinity chromatography and gel filtration. The purified protein (>95% purity) showed a single band on the gel with a molecular weight of approximately 27 kDa, similar to the calculated molecular weight of 33 kDa (the pET-28a His-tag is approximately 3 kDa). The expressed recombinant protein was detected by western blot using a 6× His mouse monoclonal antibody (**Figure 4**).

### GST Activity and Properties of RpGSTO1

**FIGURE 5:**
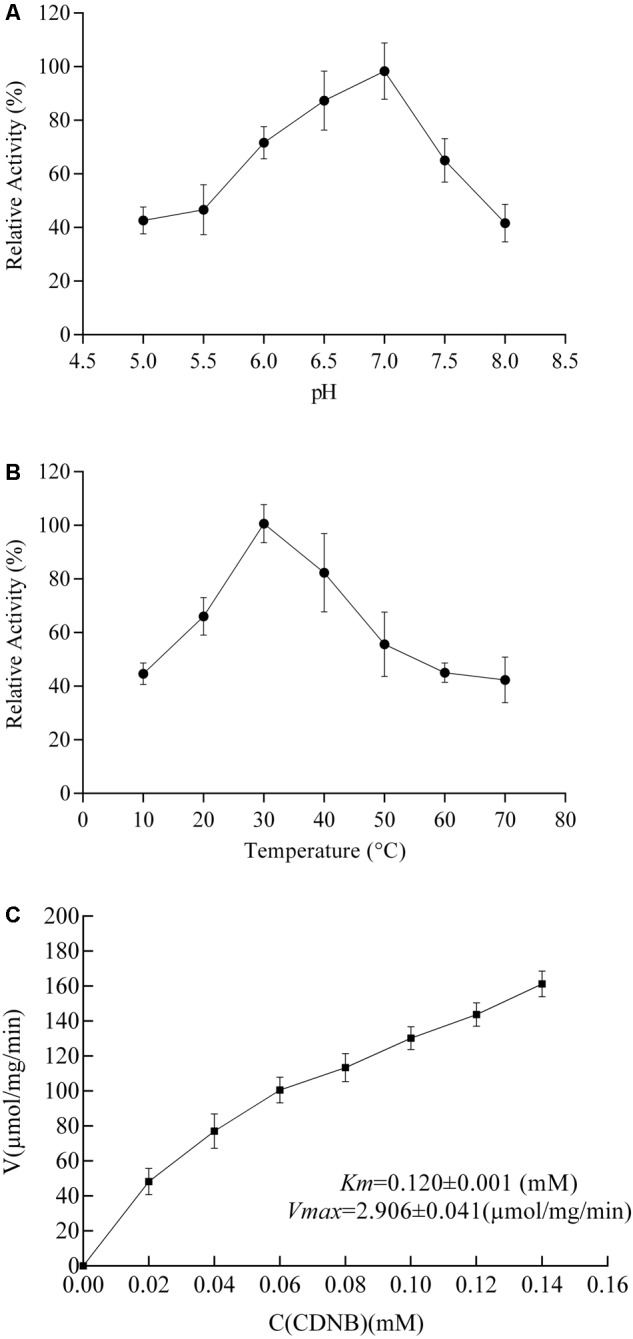
Enzymatic properties of RpGSTO1 with GSH and CDNB as substrates. **(A)** Activity of purified recombinant RpGSTO1 at various pH values. **(B)** Activity of purified RpGSTO1 activity with preincubation of the enzyme solution at different temperatures. **(C)** Activity of RpGSTO1 with different concentrations of CDNB.

The enzymatic properties of RpGSTO1 were investigated using purified recombinant RpGSTO1 with CDNB and reduced GSH as substrates. The recombinant RpGSTO1 exhibited optimum catalytic activity toward CDNB with the pH at approximately 7.0 (**Figure 5A**). The thermostability of RpGSTO1 was analyzed by measuring residual activity after incubation for 30 min at pH 7.0 and varying temperatures. The purified GST enzyme had relatively higher activity during incubation at 30°C (**Figure 5B**). Steady-state kinetic analysis was performed with 5 mM GSH and different CDNB concentrations at pH 7.0, and *K_m_* and *V_max_* were determined. Recombinant RpGSTO1 showed a *K_m_* of 0.120 mM and a *V_max_* of 2.906 μmol/mg/min (**Figure 5C**).

### Disk Diffusion Assay Performed Under Cumene Hydroperoxide Stress

**FIGURE 6:**
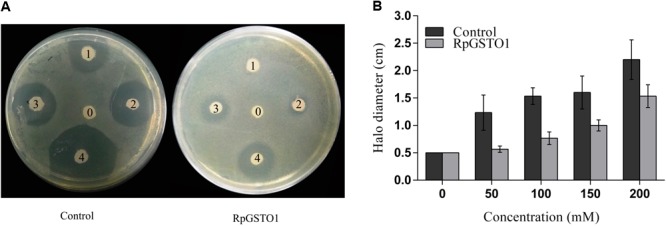
**(A)** The resistance of bacteria cells overexpressing RpGSTO1 to cumene hydroperoxide. The labels 0, 1, 2, 3, and 4 on filter disks represent different concentrations of cumene hydroperoxide (0, 30, 50, 100, and 200, respectively). **(B)** The halo diameters of the killing zones were compared in the histograms. The data are the mean ± SE of three replicates.

A disk diffusion assay was used to provide direct evidence that RpGSTO1 is capable of providing protective antioxidant activity. *E. coli* cells overexpressing RpGSTO1 were exposed to oxidative stress by treatment with cumene hydroperoxide (Burmeister et al., 2008; Liu et al., 2016). Following overnight exposure, the zones of inhibition around the cumene hydroperoxide soaked filters of the *E. coli* expressing RpGSTO1 were found to be much smaller than the control, which were transfected with the vector. The halo diameter sizes were reduced by 30% for bacteria expressing RpGSTO1 (**Figure 6**).

### Expression Profiles of *RpGSTO1* After Exposure to Different Insecticides

**FIGURE 7:**
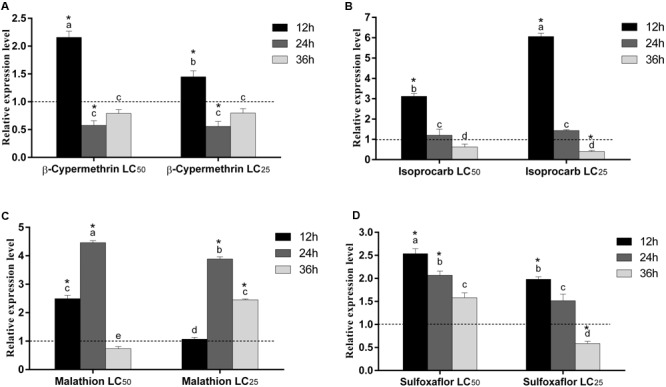
Relative expression levels of *RpGSTO1* in *R. padi* at 12, 24, and 36 h post-exposure to two different concentrations (LC_25_ and LC_50_) of four insecticides. **(A)** Exposure to β-cypermethrin; **(B)** Exposure to isoprocarb; **(C)** Exposure to malathion; and **(D)** Exposure to sulfoxaflor. Data were presented as the means (*n* = 3) ± SE. The expression level of *RpGSTO1* in the control was marked with a dash line. Different letters on the bars indicate that the means are significantly different among treatments by Tukey’s test. Asterisks above bars indicate significant differences between the treatment and the corresponding control (one-way ANOVA with Tukey’s HSD test, *p* < 0.05).

The relative expression level of *RpGSTO1* was investigated by RT-qPCR after exposure to LC_25_ and LC_50_ concentrations of β-cypermethrin, isoprocarb, sulfoxaflor and malathion (**Figure 7**). The *R. padi* were treated with LC_25_ and LC_50_ concentrations of different insecticides and the time-dependent relative expression of *RpGSTO1* normalized to their reference genes were quantified. Expression levels of *RpGSTO1* were significantly up-regulated (2.15-fold and 1.45-fold) 12 h post-exposure to the LC_50_ and LC_25_ concentrations of β-cypermethrin, respectively, compared with the untreated insect regimen. Expression levels of *RpGSTO1* were significantly lower than that of the control at 24 h and 36 h post-exposure to the LC_25_ and LC_50_ concentrations of β-cypermethrin, and the expression levels of the gene within these insecticide-treated samples were different but not statistically significant. The mRNA levels of *RpGSTO1* were significantly higher at 12 h post-exposure to the LC_25_ and LC_50_ concentrations of isoprocarb than at 24 or 36 h post-exposure. The transcription levels of the *RpGSTO1* were significantly lower at 12 h post-exposure to LC_25_ isoprocarb than that of 12 h post-exposure to LC_50_ isoprocarb. *RpGSTO1* expression was increased 4.46-fold at 24 h post-exposure to LC_50_ malathion and 3.88-fold to LC_25_ malathion, which were both significantly higher than that of 12 h and 36 h post-exposure to malathion. The mRNA level was significantly increased at 12 h post-exposure (2.49-fold) and significantly decreased at 36 h post-exposure (0.73-fold) to LC_50_ malathion, while LC_25_ doses of malathion significantly increased the expression of the gene at 36 h post-exposure. *RpGSTO1* mRNA expression level was highest 12 h post-exposure to LC_50_ and LC_25_ concentrations of sulfoxaflor. Compared to untreated insect regimen, the respective expression level of *RpGSTO1* was 2.53-fold, 2.07-fold and 1.58-fold less at 12, 24, and 36 h post-exposure to LC_50_ concentrations of sulfoxaflor, and 1.98-fold, 1.51-fold, and 0.58-fold less at 12, 24, and 36 h post-exposure to LC_25_ concentration, respectively.

## Discussion

Glutathione S-transferases are multifunctional enzymes that play a central role in the detoxification of both endogenous and xenobiotic compounds. The different classes of GST enzymes are found in a variety of insect species (Booth et al., 1961; Tu and Akgül, 2005; Li et al., 2007). The omega class of GSTs (GSTO) is a class of cytosolic GSTs with structure and characteristics that differ from other GST class (Whitbread et al., 2005; Burmeister et al., 2008). In this study, a novel GST gene of the omega class (*RpGSTO1*) was identified from the bird cherry-oat aphid *R. padi*, a serious winter wheat pest in China. A phylogenetic analysis comparing RpGSTO1 to GSTs from different classes and insects revealed that belongs into the omega class. RpGSTO1 has high identity with the GSTO1 from pea aphid *A. pisum* (**Figure 3**). The deduced protein sequence of RpGSTO1 includes conserved functional domains, including the G-site and H-site, which were highly conserved and located at the C-terminal region and N-terminal region.

The most important function of GSTs is to catalyze the conjugation of GSH to various endogenous and exogenous compounds (Hayes et al., 2005). The synthetic substrate CDNB is commonly used in GST activity assays (Ketterman et al., 2011). We observed the ability of recombinant RpGSTO1 to catalyze CDNB substrate in the presence of reduced GSH. GSTs from different insects showed high activity at different temperatures and pH values (**Figure 5**). We determined that the recombinant RpGSTO1 enzyme had optimal activity at a pH of 7.0 and a temperature of 30°C. In previous studies, the enzyme activity was stable, and high enzyme activity was observed at pH 5.0 to 8.0 from different insect GSTs (Samra et al., 2012; Yamamoto et al., 2013; Wan et al., 2016). GSTs from insects had an optimal activity at a temperature range between 25°C to 40°C (Samra et al., 2012; Zhang et al., 2013; Tan et al., 2014; Wan et al., 2016; Liu et al., 2017).

We investigated the involvement of RpGSTO1 in the oxidative stress response. To perform disk diffusion assay, we cultured *E. coli* with recombinant RpGSTO1 and the vector for a control to achieve the same cell density. Cumene hydroperoxide is a known oxidative stress inducer (Burmeister et al., 2008; Yan et al., 2013; Meng et al., 2014; Chen et al., 2015). Inhibition of the growth of the bacteria was observed following overnight exposure to cumene hydroperoxide. GSTs have a key functional role in the detoxification process involved in intracellular transport, synthesis of bio-hormones, and protection against oxidative stress of both endogenous and xenobiotic compounds (Armstrong, 1997; Enayati et al., 2005). Previous studies indicated that GSTO1 was involved in antioxidant defense (Burmeister et al., 2008; Wan et al., 2009; Yamamoto et al., 2011; Zhang et al., 2016). In this study, cumene hydroperoxide induced oxidative stress in cells expressing recombinant RpGSTO1 but showed the zone was decreased compared to cells expressing the vector (**Figure 6**). Our results provide evidence that RpGSTO1 is an antioxidant enzyme that protects cells from oxidative stress.

Insect GST can detoxify many synthetic insecticides and plant allelochemicals (Li et al., 2007). Synthetic insecticides can cause physiological changes in insects. Currently, *R. padi* has developed resistance against various insecticides (Zuo et al., 2016). To explore and characterize the putative roles that RpGSTO1 might play, we analyzed the expression patterns of the gene under different insecticide treatments (**Figure 7**). We treated insects with the pyrethroids β-cypermethrin, carbamate isoprocarb, organophosphorus malathion, and neonicotinoids sulfofoxaflor and then measured the mRNA expression level of *RpGSTO1*. The relative expression of *RpGSTO1* was affected by these insecticides, and the pattern varied among the different insecticide treatments. An omega class GST gene in *B. mori* has been reported to be induced by treatment with various environmental stresses, such as diazinon, permethrin, imidacloprid, ultra violet-B (UV-B), and bacteria (Yamamoto et al., 2011). The relative expression level of *RpGSTO1* at 12 h post-exposure to LC_50_ concentrations of β-cypermethrin, sulfoxaflor and malathion were significantly higher than the respective expression level at 12 h post-exposure to LC_25_ concentrations of each chemical, however, *RpGSTO1* expression at 12 h post-exposure to LC_50_ concentrations to isoprocarb was significantly lower that at 12 h post-exposure to LC_25_ concentrations to isoprocarb, indicating the same GSTO varied at the responses to different types of insecticides which could possibly be caused by different binding pattern of the enzyme to the chemicals. This result suggests that RpGSTO1 may play a significant role in detoxifying various groups of insecticides in *R. padi.* In previous reports, up-regulation of GST genes following exposure to pyrethroid, organophosphate, carbamate and neonicotinoid were found in insecticide-resistant strains (Hemingway et al., 1991; Yang et al., 2013; Wei et al., 2015). Down-regulation of *GSTOs* were reported in *Cnaphalocrocis medinalis* exposed to chlorpyrifos (Liu et al., 2015). GSTO gene expression was induced by different stress conditions, such as different temperature, UV, H_2_O_2_, cyhalothrin, phoxim, pyridaben, and paraquat in *Apis cerana* (Zhang et al., 2013). In this study, the mRNA level of *RpGSTO1* responded to different insecticide challenges, and the responses maybe associated with the oxidative stress caused by insecticide treatment, which were positively correlated with the previous studies, including that omega GSTs can be induced by insecticides and could play a part in detoxification of insecticides in *R. padi*.

## Conclusion

Our study demonstrated the unique functional characterization, expression pattern, and physiological roles of a novel GSTO gene from *R. padi*. To our knowledge, this is first time that an omega class GST has been cloned and characterized from the bird cherry-oat aphid. This study also revealed that recombinant RpGSTO1 possesses antioxidant activity in response to oxidative stress. The expression level of *R. padi* RpGSTO1 can be induced under the stresses caused by different insecticides. Our findings provide valuable insight into the functions of the GSTO in this serious pest.

## Author Contributions

BB and MC: conceived and designed the experiments. BB: performed the experiments. BB, KW, and MC: analyzed the data. SS and RT: contributed reagents/materials/analysis tools. BB and MC: wrote the paper.

## Conflict of Interest Statement

The authors declare that the research was conducted in the absence of any commercial or financial relationships that could be construed as a potential conflict of interest.

## Footnotes

1 http://www.ncbi.nlm.nih.gov/gorf.html

2 http://web.expasy.org/compute_pi/

3 www.graphpad.com
